# Ethnic minority carers’ experiences of in-patient mental healthcare: qualitative study

**DOI:** 10.1192/bjo.2026.12010

**Published:** 2026-05-25

**Authors:** Ella Rose, Harpreet Gill, Alice Wickersham, Lisa Wood

**Affiliations:** Division of Psychiatry, University College London, UK; Hospital Division, North London NHS Foundation Trust, London, UK

**Keywords:** Carers, in-patient treatment, qualitative research, patients and service users, thematic analysis

## Abstract

**Background:**

Ethnic minority groups are disproportionately represented in in-patient mental health services, yet research has largely overlooked their carers’ experiences. Because carers play a crucial role in managing their family member’s mental well-being, gaining insight into their potentially unique challenges and support needs is essential for service development.

**Aims:**

To investigate the experiences of ethnic minority carers of patients receiving in-patient psychiatric care in the UK, identify challenges they face and understand the role of culture and ethnicity in an in-patient setting.

**Method:**

In the UK, ‘ethnic minority’ refers to all individuals from a non-White British background. Twelve carers from ethnic minority groups were recruited from third-sector organisations across the UK to participate in semi-structured interviews online. Audio-recordings of the interviews were transcribed, and data were analysed with reflexive thematic analysis.

**Results:**

Three core themes were identified: ‘The Challenges of Navigating Hospital Admission’, ‘Cultural and Carer Exclusion in in-patient Care’ and ‘Improving the Ethnic Minority Carer Experience’. Findings elucidated the nuanced ways in which culture and ethnicity influence carers’ experiences. Participants highlighted systemic barriers to timely support, experiences of disempowerment and exclusion, and culturally insensitive care. Participants offered recommendations to improve support for carers.

**Conclusions:**

Ethnic minority carers of in-patients experience difficulties arising from a lack of cultural safety within in-patient psychiatric services. Embracing a holistic approach to care and providing comprehensive, culturally responsive guidance and support for carers are essential for promoting equitable mental healthcare.

The most extensive data on ethnic inequalities in mental healthcare come from the UK, where disparities in access, experiences and outcomes have been documented for over 50 years.^1^ In the UK, the term ‘ethnic minorities’ is often used to refer to all ethnic groups other than White British.^2^ Compared with their White counterparts, ethnic minorities are disproportionately diagnosed with severe mental illnesses^3^ yet are less likely to be referred to mental health services.^4^ They face a significantly higher likelihood of involuntary detention under the Mental Health Act:^5,6^ recent rates for Black and Black British individuals were three and a half times higher than those for White British individuals.^7^ Consequently, ethnic minority groups are overrepresented in in-patient psychiatric care,^8^ often facing longer stays and higher readmission rates.^1^ These inequities persist^9^ despite cross-government initiatives (e.g. the National Health Service (NHS) Long Term Plan,^10^ Reforming the Mental Health Act^11^ and the Patient and Carer Race Equality Framework^12^). However, ethnic minority experiences of in-patient mental healthcare, and those of the individuals who support them (hereafter referred to as ‘carers’), remain largely overlooked in research.

Carers are a vital resource to UK health services; the economic impact of their contribution alone is estimated at £162 billion, surpassing the total budget of NHS England.^13^ Of the estimated 7 million unpaid carers in the UK, 1.5 million support individuals with mental health issues.^14^ They play an integral role in managing the long-term mental well-being of their family member, often at great personal cost,^15^ and the shift towards deinstitutionalisation and community-based care has resulted in a heavy reliance on their unpaid support.^16^ Carer involvement in in-patient settings has been linked to improved outcomes,^17^ including reduced relapse hospitalisations and shorter stays.^18–20^ Carers and service users prefer services that include and work alongside carers.^21^ Accordingly, the UK has developed guidelines and policies, such as the National Carers Strategy^22^ and The NHS Triangle of Care,^23^ to support and engage carers effectively. However, in in-patient services, the notion of carers as ‘equal partners’ in care often falls short. Despite their expertise and willingness to be involved,^24^ carers worldwide report negative experiences^25^ citing poor communication, barriers to collaboration and a lack of emotional and practical support.^26–30^

Research on carers’ experiences of mental health services is growing, yet there is a notable lack of diversity within the existing literature, particularly in in-patient settings.^27,29,31,32^ Systematic disadvantages persist for certain groups in the UK: individuals from ethnic minority backgrounds report significantly more negative perceptions and experiences of mental healthcare.^33^ Hence, ethnic minority carers, although facing the universal challenges associated with caregiving, may also encounter unique difficulties in in-patient services. Indeed, ethnic disparities are evident from the pathway to admission. In particular, Black service users and, as a result, their carers, often experience more interactions with the police and the criminal justice system.^34^ However, studies show that this increased police contact does not align with worse functioning or illness severity.^35^ On in-patient wards, Black service users are also more likely to be placed in seclusion or restrained.^36^ Recent qualitative research described Black carers’ interactions with in-patient services as a ‘battle’.^37^ A lack of cultural sensitivity in mental health services has been well documented; carers frequently encounter stigmatisation, discrimination, language barriers and stereotyping, cultivating a distrust of mental health services.^38^

Most literature does not focus on carers’ experiences within in-patient environments, or else combines service user data with those of carers,^25,39^ thereby neglecting to explore carers’ viewpoints in depth. This qualitative study aims to address this gap by examining the experiences of ethnic minority carers for patients receiving in-patient mental healthcare in the UK. By amplifying marginalised voices in research and practice, this study contributes to the broader discourse on carer support. Understanding diverse perspectives is crucial for developing equitable, responsive mental health services that improve engagement and satisfaction.

## Method

### Design and ethics

This qualitative study used semi-structured interviews with carers belonging to ethnic minority groups with family members who have been admitted to mental health in-patient settings. The authors assert that all procedures contributing to this work comply with the ethical standards of the relevant national and institutional committees on human experimentation, and with the Helsinki Declaration of 1975 as revised in 2013. The study was approved by the University College London (UCL) Research Ethics Committee (no. 27265/001). Written informed consent was obtained from all participants. The study is reported according to the Consolidated Reporting for Qualitative Research (COREQ) guidelines.

### Recruitment

Participants were recruited through convenience sampling by voluntarily responding to a flyer advertising the study, distributed via the research teams’ professional networks, social media and representatives of third-sector organisations that provide support for carers whose family members have mental health difficulties. Participants were not known to the research team.

Twelve ethnic minority carers were recruited, meeting the recommended minimum required to reach data saturation. No participants dropped out once consented into the study. Eligible participants belonged to an ethnic minority group, as defined by GOV.UK^2^ to include ‘all ethnic groups except White British’, including White minority groups such as Gypsy, Roma and Irish Traveller. Participants were carers of someone who is currently, or had been admitted to, in-patient mental healthcare in the UK within the past 3 years. This was established to ensure the relevance of their experiences within the current landscape of in-patient services. Excluded were those under 18 years, those without sufficient command of English to consent or participate without translation, and those unable to give informed consent.

### Procedure

All participants were interviewed by the primary author (E.R., MSc student). Interviews were conducted remotely one-to-one and recorded via video-conference, either on Zoom or Microsoft Teams. E.R. liaised with participants via telephone or email to arrange the interviews, and met with them for a single interview session to undertake the interview.

Interviews followed a preprepared topic guide (see supplementary material available at https://doi.org/10.1192/bjo.2026.12010 for primary questions), developed in consultation with two clinical psychologists who have experience of working in in-patient care and with carers. The guide was constructed to reflect themes from the existing literature, and adapted to explore their relevance to ethnic minority carers. The guide focused on three key areas: (a) navigating in-patient psychiatric care, (b) the role of their culture or ethnic identity and (c) support received and suggestions for improvements.

Interviews were audio-recorded with participants’ consent. Participants were informed about E.R.’s reasons for undertaking the research. Interviews were transcribed verbatim by E.R., omitting any personal data, and each participant was assigned a numeric code. All data were obtained and stored in accordance with UCL Data Protection policies. Interviews lasted between 20 and 65 min, with a mean length of 46.5 min. Participants did not receive transcripts of the interviews.

### Analysis

Reflexive thematic analysis was used, following the six-step analytic procedure outlined by Braun and Clarke.^40^ Analysis was conducted by E.R. and facilitated by NVivo 15 for macOS (Lumivero, Denver, Colorado, USA; https://lumivero.com/products/nvivo/). The transcribed data were re-read several times, and an inductive approach was employed to code the transcripts line-by-line at a semantic level, focusing on explicit, surface-level data related to the research question. Codes were grouped into broader categories and then developed into potential themes. Codes were organised into themes through an iterative process and reviewed by the research team to ensure clarity and depth. Once the themes and their interconnections were established, they were defined and labelled. In the final step, a detailed report was produced. Participants did not provide feedback on the findings.

### Research reflexivity

E.R.’s experience as a carer informed her interest in this research. As a White British female researcher, she engaged in ongoing reflexive practice and critical consideration of her positionality to ensure that interpretations were thoughtful and sensitive. The research was supervised by L.W. and H.G., who both have extensive experience working in mental health hospital settings and conducting research with patients and carers from ethnic minority backgrounds. The team recognises the well-documented inequalities affecting ethnic minority carers within UK mental health services, including disparities in access and experiences of coercion and outcomes, and remained mindful of how this context might shape the research. E.R., who led the analysis, kept reflective notes throughout the study and considered how her background, assumptions and professional identity might influence interpretation. Analytical decisions were discussed regularly with L.W. and H.G. to ensure that findings remained grounded in participants’ accounts.

## Results

A total of 12 participants took part in the study. Most participants were female (83.3%) and parents or siblings to their family member who had used in-patient mental health services. Participants had a mean age of 47.75 years (s.d. = 14.21 years). Two participants were from a White minority background, three from Asian or Asian British backgrounds, three from Black British, Caribbean or African backgrounds and four from mixed or multiple ethnic backgrounds. The most common diagnosis of their family member was depression (*n* = 4), followed by anorexia nervosa (*n* = 3), schizophrenia (*n* = 3) and bipolar disorder (*n* = 2). The majority of family members were admitted compulsorily to the in-patient ward. Table 1 presents the demographic characteristics of the participants.

**Table 1:** Demographic characteristics of sample

Participant demographics/characteristics	*N* (%)
Age, years: mean = 47.75 (s.d. = 14.2; range = 30–73)	
Gender	
Female	10 (83.3)
Male	2 (16.7)
Ethnicity	
White minority background	2 (16.7)
Italian	1
Jewish	1
Asian/Asian British	3 (25.0)
Indian	2
Pakistani	1
Black/Black British, Caribbean or African	3 (25.0)
Caribbean	2
Caribbean and African	1
Mixed/multiple-ethnic background	4 (33.3)
White and Black Caribbean	1
Other mixed/multiple ethnic	3
Arab, Italian and Algerian	1
Indian, South American, Indo-Guyanese, Caribbean, Middle Eastern	1
Latin American, Arab, Traveller	1
Employment status	
Full-time	5 (41.7)
Part-time	3 (25.0)
Retired	3 (25.0)
Unemployed	1 (8.3)
Marital status	
Married	6 (50.0)
Single	5 (41.7)
Separated	1 (8.3)
Religious beliefs	
No religion	5 (41.7)
Muslim	4 (33.3)
Christian	2 (16.7)
Jewish	1 (8.3)
Relationship to service user	
Parent	4 (33.3)
Sibling	4 (33.3)
Son/daughter	2 (16.7)
Partner	1 (8.3)
Son/daughter-in-law	1 (8.3)
Service user diagnosis	
Depression	4 (33.3)
Anorexia nervosa	3 (25.0)
Schizophrenia	3 (25.0)
Bipolar disorder	2 (16.7)
Admission type	
Compulsory	9 (75.0)
Voluntary	3 (25.0)

### Thematic analysis

Three superordinate themes were generated: ‘The challenges of navigating hospital admission’, ‘Cultural and carer exclusion in in-patient care’ and ‘Improving the ethnic minority carer experience’, comprising ten subordinate themes (presented in Table 2).

**Table 2:** Superordinate and subordinate themes

The challenges of navigating hospital admission	Barriers and dismissal in the pathway to admission
	The emotional impact of admission
	A confusing system to navigate
Cultural and carer exclusion in in-patient care	A lack of culturally sensitive care
	Exclusionary communication: barriers to information, involvement and accountability
	Exclusion of carer expertise from care
	Marginalised and misunderstood
Improving the ethnic minority carer experience	Culturally informed and accessible carer support
	Embracing culture on the ward
	Advice for other carers

### The challenges of navigating hospital admission

Before admission, carers recalled the challenges of securing timely support for their family member and the difficulties in navigating a distressing and complex process with minimal guidance.

#### Barriers and dismissal in the pathway to admission

The lead-up to in-patient admission was marked by stress and barriers to accessing timely and appropriate support for their family member. Participants recounted dismissive responses from services: help seemed available only once crises arose. Interactions with crisis teams and emergency psychiatric services were repeatedly characterised as unhelpful or lacking in empathy. Long waits for psychiatric bed spaces exacerbated distress, leading some to take it upon themselves to find suitable facilities.

Several participants reflected on how limited mental health awareness in their ethnic and cultural communities impacted their ability to recognise early warning signs. Some struggled to reconcile their cultural beliefs with the severity of their family member’s condition and the need for treatment: ‘my mum would be like, “there’s nothing wrong with him”, because it’s within our culture to just… cover our problems’ (Participant 8 (P8)).

Whereas most participants viewed admission as warranted, some perceived racial prejudice as contributing to unnecessary admissions:

‘I said to the mental health team, “he’s not that unwell, he doesn’t need admission, what he needs is support in the community”…that is just dismissed… “He’s a very dangerous young black man. So, he needs to be in hospital”, so he’s admitted… do you hold everybody by those same standards, or is it because you’re faced with this person who fits a stereotype?’ (P10)

#### The emotional impact of admission

All participants reported that admission affected their own well-being, with over half explicitly referring to the experience as ‘traumatic’, resulting in a variety of negative emotional responses. Distress stemmed from the tumultuous admission process and the emotional strain of caring for a family member whose condition had significantly deteriorated, with some likening it to grief.

Several participants noted that the emotional toll of their family member’s admission was intensified by stigma in their cultural communities, leading to feelings of shame that, in some cases, impacted multiple generations of family members: ‘In the Asian society… it’s shameful… to say that somebody’s got mental illness or disorder’ (P1).

Moreover, any initial relief post-admission appeared short-lived due to negative preconceptions of in-patient care. This resulted in fear and anxiety due to ongoing concerns for their family member’s safety. Hospitals were portrayed as intimidating and unwelcoming places for carers. Relinquishing care of their family member to a setting they viewed with scepticism was a source of significant anxiety:

*‘*These places are often called places of safety, but I can’t correlate that to the environments that I’ve seen… you’re constantly thinking… have I left them in the right place?… There’s constant worry’. (P10)

#### A confusing system to navigate

Participants consistently noted a lack of guidance and practical support. Many felt inadequately informed about ‘basic’ aspects of their family member’s care. Participants wanted more information on diagnoses, treatment options and medication; most had to conduct their own research to feel equipped to cope with the situation. In-patient stays ranged from 1 week to 2 years. Seven participants reported multiple admissions. A gradual learning curve emerged:

*‘*We just have to figure it out ourselves… it’s like the more you do it, the better you get… which is really sad’. (P8)

This lack of support left participants with numerous unanswered questions and no clear guidance on where to obtain answers, fostering distrust in the system:

*‘*They don’t inform you of anything extra, or anything that you should or could be aware of, whether it’s rights, what you’re entitled to, or whether it’s the support that you could get… they hide things’. (P1)

### Cultural and carer exclusion in in-patient care

This theme explores carers’ predominantly negative experiences of in-patient care, chiefly the approach to their family member’s treatment, communication issues and a prevailing sense of disempowerment and exclusion.

#### A lack of culturally sensitive care

Although some participants acknowledged a positive effect of hospitalisation on their family member’s recovery, only one explicitly described an in-patient facility as *‘*very good’ (P2). The majority of participants expressed concerns regarding a lack of person-centred care that took into account their family member’s cultural background. Participants advocated for a more holistic approach:

*‘*Cultural nuance is important… see the person not just as the illness, but also their cultural background… not just a clinical approach’. (P11)

They expressed disappointment over the lack of psychotherapy, therapeutic activities, proactive staff engagement and alternative treatment modalities. Many were wary of the medication-focused approach to treatment, which created tension between carers and professionals:

‘My mum’s Rasta, we grew up with a very natural way of living… [the hospital] were just saying so much information about medication… for my little brother, who doesn’t even take paracetamol… [he said] can we not do this holistically?… the holistic word– saying that in a hospital is almost, like, “what are you talking about? Like, just sit down. Be quiet. Maybe you shouldn’t be part of the plan”’. (P8)

Frustration was attributed to the quality of staff. Whereas participants valued interactions with staff who showed genuine care and connection, they encountered staff who seemed disrespectful or apathetic in their roles. Issues were raised about cultural awareness among staff, the lack of consideration of personal care, dietary restrictions and religious practices:

*‘*My mum’s care – having an afro, for example… knowing how to care for her skin. Her skin is much drier; her hair is much drier… I don’t even have the words to describe how she’s being treated because I can see it’s different’. (P9)

‘Practising your faith, I think this is a limitation in the hospital… I’m not saying he wasn’t allowed, but there was some sort of pressure… they don’t really understand. It is a comfort, you know, a way of finding their peace… they shouldn’t be restricted’. (P3)

Other participants noted that minimal effort was made to accommodate carers’ cultural needs, such as amending leave hours for religious holidays. This disregard was disconcerting, because they felt that honouring these practices fostered a sense of normalcy for them and their family member. Although participants valued ethnic diversity in the wards, some voiced concerns that the formation of *‘*cliques’ among staff, based on shared languages or cultures, undermined the therapeutic environment and alienated their family member.

#### Exclusionary communication: barriers to information, involvement and accountability

Clear and consistent communication emerged as the primary need for most participants; however, nearly all reported feeling insufficiently informed, with staff failing to provide regular updates on their family member’s progress, thereby contributing to significant anxiety. Participants wanted proactive updates rather than having to actively pursue this information: ‘It became a full-time job… the emails, the phone calls, it was every day… it affected my mental health’ (P1). Additionally, participants highlighted that, in some cultures, it is not customary to demand things, which made this aspect of in-patient care particularly challenging.

The conflict between carers’ need for information and patient confidentiality led to discontent. Carers argued that their inability to stay informed during critical moments jeopardised their family member’s safety. This issue was particularly pronounced when it involved in-patients who were not fluent in English; carers explained that their family member might not fully comprehend the implications of consent, particularly with inadequate translation services. There was some perception that stereotypical prejudgements further hindered communication: for example, one participant described how her mother-in-law wearing a Niqab was perceived to be a barrier:

*‘*There are so many biases… my mother-in-law wears the full veil, the Niqab, that covers her face… healthcare professionals, they think this is a barrier of interaction with her’. (P3)

Mishandling of complaints surfaced as a major grievance for 10 out of the 12 participants. There was often no clear avenue for providing feedback or voicing concerns. Furthermore, where participants felt vulnerable due to their ethnicity, they hesitated to raise issues:

‘Being a non-White person, I wasn’t sure if I raised anything, whether that would have repercussions for my sister, so I don’t think I brought anything up’. (P5)

Those who lodged complaints reported that their concerns were often acknowledged only after prolonged delays or were not addressed at all, even after escalating issues to higher hospital authorities.

#### Exclusion of carer expertise from care

There was universal dissatisfaction with the level and quality of involvement experienced by carers. Participants frequently found their input disregarded. Some reported being actively excluded from care-planning or facing structural barriers that prevented meaningful participation. Others contended that their contribution was superficially acknowledged, referring to the use of *‘*buzz words’ and ‘tick box exercises’. This tokenistic engagement led to a perceived lack of agency, with participants discerning a significant power imbalance between themselves and the professionals: ‘The doctors, whoever was in authority, they came across like they knew better, and that was how it was gonna be’ (P7).

Nevertheless, many feared that critical aspects of their family member’s care would be neglected or mishandled without their active involvement because they held unique and valuable knowledge about their family member that in-patient staff did not. They stressed the importance of their role as a reliable source of support and advocacy:

*‘*When he gets into hospital… the way he speaks is interpreted differently to others… if you’d invited me to the ward round, I could have, you know, translated the street talk. It’s something that you wouldn’t understand’. (P10)

#### Marginalised and misunderstood

Although not all participants could articulate exactly how their ethnicity and culture influenced their experiences, 8 out of the 12 believed they were treated differently. Several suspected prejudice among hospital staff, suggesting that biases affected their interactions and involvement in their family member’s treatment:

*‘*Being a Muslim, visibly wearing a scarf… people automatically assume that I don’t understand things or can’t articulate myself or can’t ask for information… that is amplified in places like wards and hospitals, where there are formal processes… people will feel like you can’t engage’. (P4)

Some felt that staff probed for cultural or religious explanations behind the aetiology of their family member’s illness, which they interpreted as shifting blame onto carers.

Differential treatment could be subtle, planting seeds of doubt about the fairness of their treatment. This was often expressed as micro-aggressions, conveyed through staff’s body language or tone, leaving participants uneasy and insecure:

‘You could just see – people will ignore you or they’ll talk down to you… it’s the whole body language thing… you’ll question yourself and say, “was there something I did?”… and then eventually you say, “well, what is it about me?” And that’s when you start looking in the mirror’. (P10)

Some examples were more overt:

*‘*They asked if my wife and I were related… and if [daughter] was going to have an arranged marriage… it did seem to be almost as if we were, you know, at times maybe frowned upon’. (P1)

Participants, particularly those from Black ethnic groups, highlighted how systemic discrimination, racism and generational trauma shape experiences with mental health services. They expressed concerns that mental health professionals often overlook intersectional factors and their influence, leaving key aspects of their identities and cultural histories unrecognised. Many described their identities as deeply intertwined with mental health, yet these dimensions were misunderstood or ignored:

‘I don’t know if the NHS is culturally sensitive and aware of what Black people experience… trauma is very much part of our history… it’s part of our everyday lives’. (P8)

Some participants from mixed ethnic backgrounds echoed these concerns, particularly around the issue of *‘*mixed-race erasure’ (P6).

### Improving the ethnic minority carer experience

This theme illustrates the shared changes they wanted in in-patient services and advice they had for other carers.

#### Culturally informed and accessible carer support

All participants wanted reassurance that their family member was safe and receiving the treatment they needed. They believed this required proactive efforts to engage carers, and better written and verbal communication, with translation available when necessary.

Participants wanted established carer support systems; others proposed: *‘*peer systems’ (P8), whereby experienced carers could guide new carers through the in-patient system. Due to fear of stigma, participants also advocated for more structured opportunities to connect and share their challenges with carers from similar cultural backgrounds without judgement:

*‘*For people who are from an ethnic minority background, there’s still a lot of stigma associated… you will not admit to, you know, to other family or friends that you’ve got this sort of illness… the fact that there is this issue for many people is important’. (P11)

Many participants articulated a need for psychological support for themselves, including culturally sensitive family therapy, ideally from practitioners from similar backgrounds who appreciated the nuanced dynamics within their families. At a minimum, they expected staff to show genuine concern for their well-being:

*‘*All staff should have a responsibility to acknowledge the carers… show some genuine curiosity about how you’re doing… it is such a stressful thing to have to deal with’. (P10)

Some participants suggested a *‘*carer’s kit’ (P2), that might provide comprehensive written information, available in multiple languages, on their family member’s treatment plan and prognosis, hospital processes, responsible staff and available services or self-care tools for carers. This need was considered particularly pertinent for families with limited English: *‘*If you’re not fluent in English it’s a very real, real challenge’ (P3).

A few participants shared that they did not initially identify as ‘carers’, viewing their role as a familial duty rather than something that entitled them to support. They felt that earlier recognition would have allowed them to access support sooner:

‘At the beginning I was not identifying myself as a carer… in certain cultures there should be more education about care and responsibility… there is support out there’. (P3)

#### Embracing culture on the ward

Participants unanimously acknowledged that their culture and ethnicity influenced their experiences in in-patient mental healthcare, albeit in nuanced ways. By approaching patients and carers with genuine curiosity and humility, participants believed staff could cultivate trusting relationships.

Participants shared ideas for fostering inclusive and therapeutic environments for ethnic minority service users and their carers. Many highlighted the positive role of culture in recovery:

*‘*For us, culture was something nice to bring her back into. It was something to try and have her look forward to’. (P2)

Incorporating culturally inclusive activities, such as yoga or Ayurveda, or creating opportunities to connect with family members through cultural practices such as preparing and sharing traditional foods, were proposed to provide holistic support. Some participants recommended inviting organisations that advocate for ethnic minority mental health to give talks on the ward, offering avenues of support post-discharge. Thoughtful redesign of the ward space to reflect its diverse users was suggested; one participant was pleased to find a prayer room in a facility that featured artwork and religious materials from multiple cultures.

Some questioned whether cultural sensitivity could be achieved through training alone, fearing that ingrained biases would persist despite policies or guidelines:

*‘*You could have the most beautiful policies, but if we cannot make people accountable… it’s a waste of time… all the policies and the learning experiences and the workshops… it’s a joke’. (P12)

Overall, participants expressed a desire for real, tangible change.

#### Advice for other carers

Participants emphasised the need for assertiveness and persistence in advocating for their family member. They agreed that navigating the in-patient system as an ethnic minority carer requires resilience:

*‘*We’ll be fighting ‘til the cows come home… I don’t know how anyone’s had an easy, smooth ride in getting what they want… the worst thing about it is you’re not even asking for a luxury item. You’re asking just for your loved one to be cared for’. (P9)

Reflecting on the emotional toll of caregiving, they advised making space for self-care and spoke about the importance of maintaining healthy boundaries with their family member. Isolation was a common concern; participants noted the value of a strong support network.

Multiple participants were actively involved in research, organisations and carer groups. Some voiced disappointment over the lack of representation of ethnic minority carers in research, recognising that their lived experience offers valuable insight for service development, and encouraged others to share their feedback in pursuit of positive change.

## Discussion

This study provides insight into the underresearched experiences of ethnic minority carers of patients receiving in-patient psychiatric services. Participants described lack of timely support, traumatic admissions and pervasive distrust of services, compounded by inadequate guidance and support. Dissatisfaction with care approaches, poor communication and exclusion from their family member’s care reinforced perceived power imbalances with professionals. Culture and ethnicity impacted these experiences in nuanced ways. Instances of cultural insensitivity, differential treatment and limited mental health awareness within some communities were discussed. Participants proposed changes to in-patient care, positioning this study as a valuable contribution to service development.

Concerns about quality of care, poor communication, barriers to involvement and insufficient support during hospitalisation have been well documented by carers,^27,30^ irrespective of ethnic or cultural background, underscoring enduring challenges. However, a distinctive finding is that all 12 participants reported their ethnicity or culture as influencing their experiences of in-patient psychiatric care.

Previous studies frequently characterise ethnic minority experiences of mental health services as a ‘battle’,^1^ a sentiment echoed by our participants. Difficulties in obtaining support are not unique to this group, nor to the UK. Bartl et al^25^ identified a global trend in which carers’ concerns are often dismissed until hospitalisation becomes unavoidable. Ethnic minority groups are more likely to engage with mental health services for the first time during a crisis, making negative perceptions of crisis teams and emergency psychiatric services concerning, especially given their prioritisation in the UK’s mental health reform agenda (e.g. The Crisis Care Concordat; The Five Year Forward View for Mental Health). Participants also described a lack of awareness or acceptance of mental illness within their communities, which can hinder help-seeking.^34^

Our findings align with those of Miller et al,^38^ who reported criticism of mental health services for a lack of cultural awareness. Although differential treatment based on ethnicity has been well documented among in-patients,^34^ this study delineates both overt and subtle instances of such treatment towards carers. Participants from Black and mixed-ethnic backgrounds wanted professionals to recognise the role of racism and discrimination in mental illness, and their experiences. Similarly, Ajala^37^ identified concerns among Black carers about the neglect of intergenerational trauma in mental health treatment.

Participants wanted a more holistic approach to care. Although there is evidence that faith and spirituality support recovery,^34^ several participants felt that their religious practices were misunderstood or ignored by professionals.

### Strengths and limitations

This study adhered to COREQ best-practice guidelines for qualitative research. By limiting experiences to the past 3 years, these findings depict both recent and ongoing challenges. Our recruitment approach resulted in a diverse group with various caring roles, with family members both voluntarily and compulsorily admitted to different in-patient services (e.g. child and adolescent, adult, psychiatric intensive care unit, acute) across the country, with varied lengths and numbers of stays. In line with GOV.UK^2^ guidelines, we included White minority carers, recognising the disparities they also face in mental healthcare. The consistency of participants’ accounts suggests that the challenges identified are widespread across multiple ethnic backgrounds, highlighting critical areas for improvement.

Only 12 carers were sampled, the majority of whom were female. Only one partner was included. The idiographic nature of qualitative research supports in-depth analysis from smaller samples, although the convenience recruitment strategy, largely through third-sector organisations, may limit generalisability. We did not have the required ethics approval to recruit directly from NHS organisations, which may mean that our sample is not reflective of current NHS in-patient carers. Many participants were involved in policy, research or advocacy, introducing potential selection bias.

Ethnic minority carers were not involved in developing the interview schedule, and interviews were conducted exclusively in English. Language barriers hinder engagement with mental health services,^33^ and this study may not reflect the experiences of carers who face such challenges. Moreover, although combining ethnic groups probably resulted in important nuances being omitted, this approach was appropriate to enhancing the diversity of carers’ perspectives within the literature.

A further potential limitation concerns the use of reflexive thematic analysis, because a phenomenological approach may have offered greater depth in exploring individual participant experiences. Nonetheless, reflexive thematic analysis was considered appropriate for addressing the aims of the study.

### Implications and future research

To better empower carers, cost-effective solutions such as ‘carers kits’ with essential information in multiple languages could be beneficial. Some participants did not initially identify as ‘carers’, and services should thus be aware that this can prevent some carers from seeking support for themselves. Formal guidance from carers with similar experiences could help ethnic minority carers feel more seen and supported. Roles such as carer champions and family ambassadors have demonstrated positive outcomes and could represent an important source of support and advocacy. Ethnic minority carers should help inform training for these roles. in-patient services should also consider offering culturally adapted family therapy, which has shown promise in previous research.

Stigma discouraged some participants from seeking support within their social networks, leaving them feeling alienated. Staff should be trained to signpost carers to appropriate organisations, such as ethnic minority carer support groups, which may offer protective social networks. Partnerships with third-sector organisations could extend support beyond discharge.

Participants struggled to feel heard in in-patient settings. Our findings indicate that carers would welcome safe spaces in which they are appreciated as experts, to share their feedback. Future research on carers within in-patient settings must amplify the views of minoritised communities to ensure more equitable service provision. Further qualitative research should focus on carer experience of in-patient care with specific ethnic minority groups to ensure that the nuance of experience is captured. Future studies should prioritise larger, diverse samples to determine whether the themes identified in this study are applicable across various backgrounds. In particular, the experiences of young carers, as well as those of refugees and asylum-seekers, warrant exploration.

Although ethnic minority carers face challenges similar to those faced by all carers within in-patient mental health services in the UK, their difficulties are often amplified. Prioritising culturally responsive, holistic care that meets the multidimensional needs of ethnic minority carers, and their family members, is essential to addressing inequalities within the system.

## Supporting information

10.1192/bjo.2026.12010.sm001 Rose et al. supplementary material Rose et al. supplementary material

## Data Availability

The data that support the findings of this study are available from the corresponding author, L.W., upon reasonable request.

## Supplementary material

The supplementary material is available online at https://doi.org/10.1192/bjo.2026.12010
