# Supplementary material for: Ethnic minority carers’ experiences of in-patient mental healthcare: qualitative study
Source: BJPsych Open. 2026 May 25;12(3):e148. doi: 10.1192/bjo.2026.12010 (PMC13202605; doi:10.1192/bjo.2026.12010)
Supplement: Rose et al. supplementary material [file S2056472426120109sup001.docx]

**Interview Topic Guide**

1. **Perceptions/ experiences of inpatient mental health care**

- Before your family member was admitted into hospital, how well did you understand the process of admission into inpatient mental health care?

1. **Carer involvement in inpatient mental health care**

- Did you have as much involvement in developing your family member’s care plan as you would’ve liked?

1. **Communication with hospital staff**

- Can you describe what your communication is/was like with staff on the ward?

1. **Support for carers**

- Can you tell me about any support you have been offered by health professionals while your family member has been/was in hospital?

1. **Recommendations to improve support for carers**

- Can you think of any changes to inpatient services that you think are necessary to provide better support for carers?
